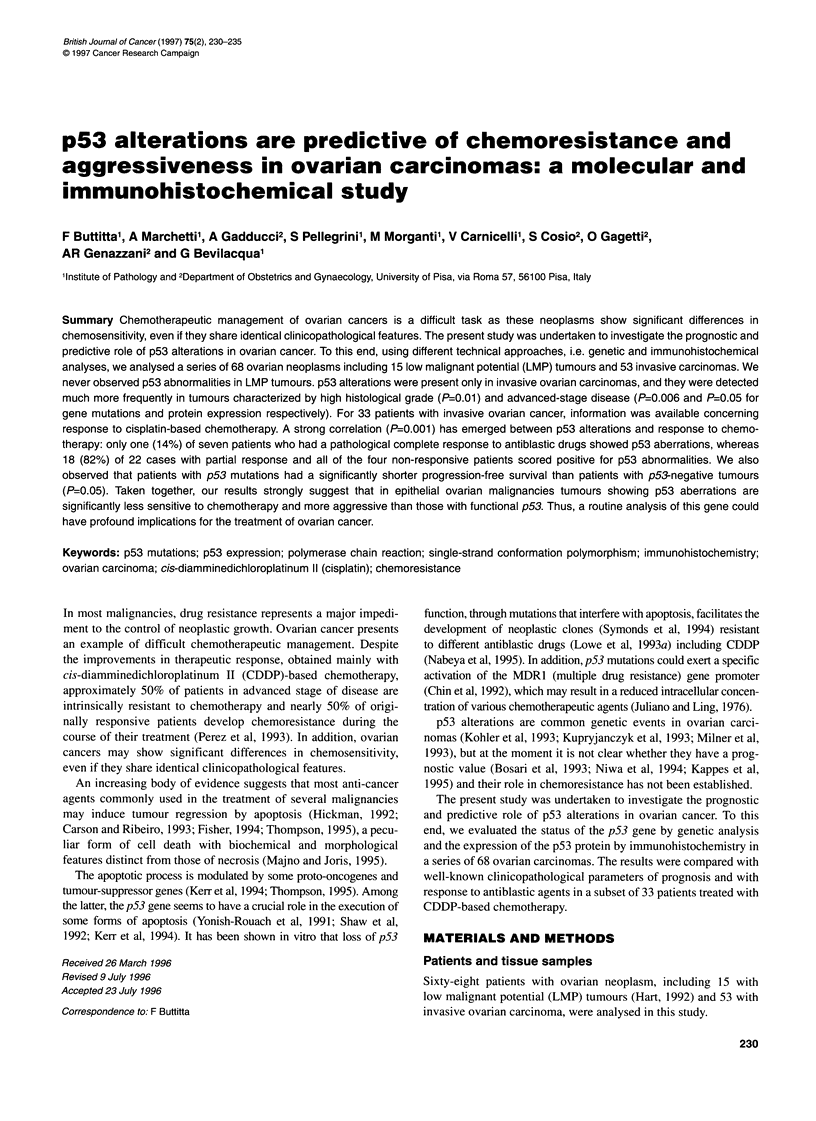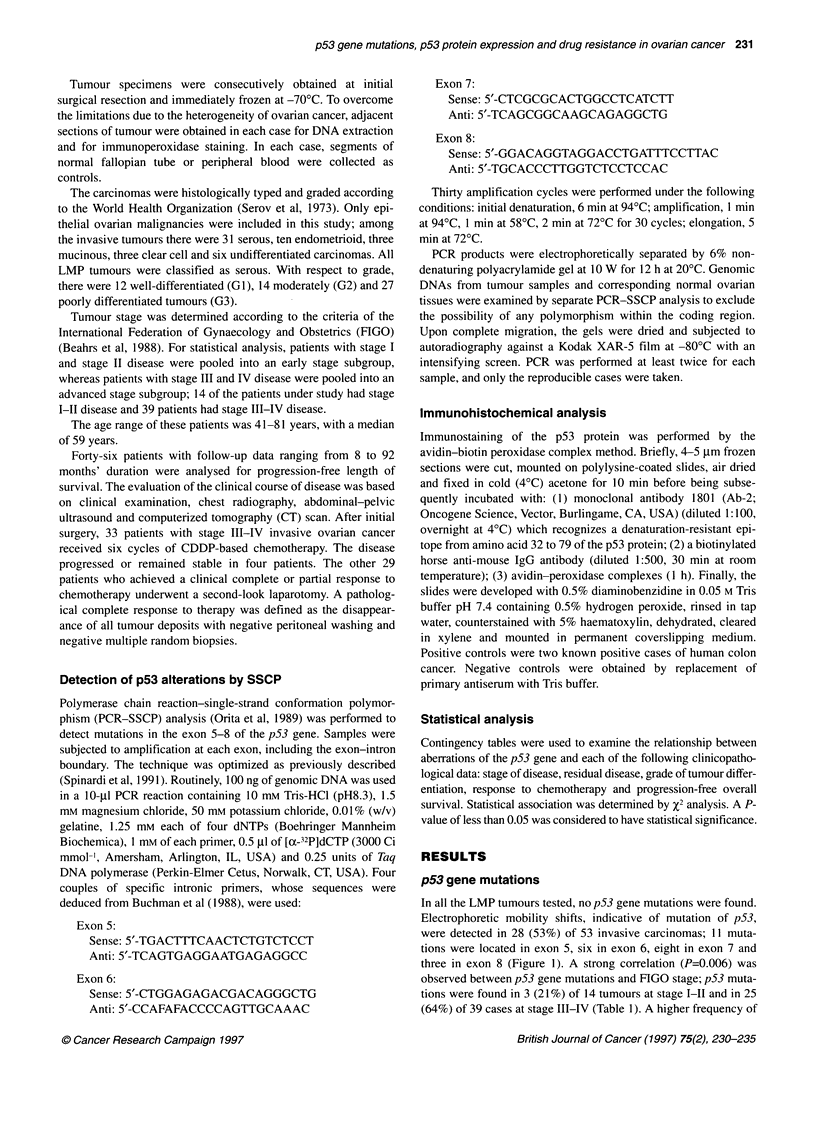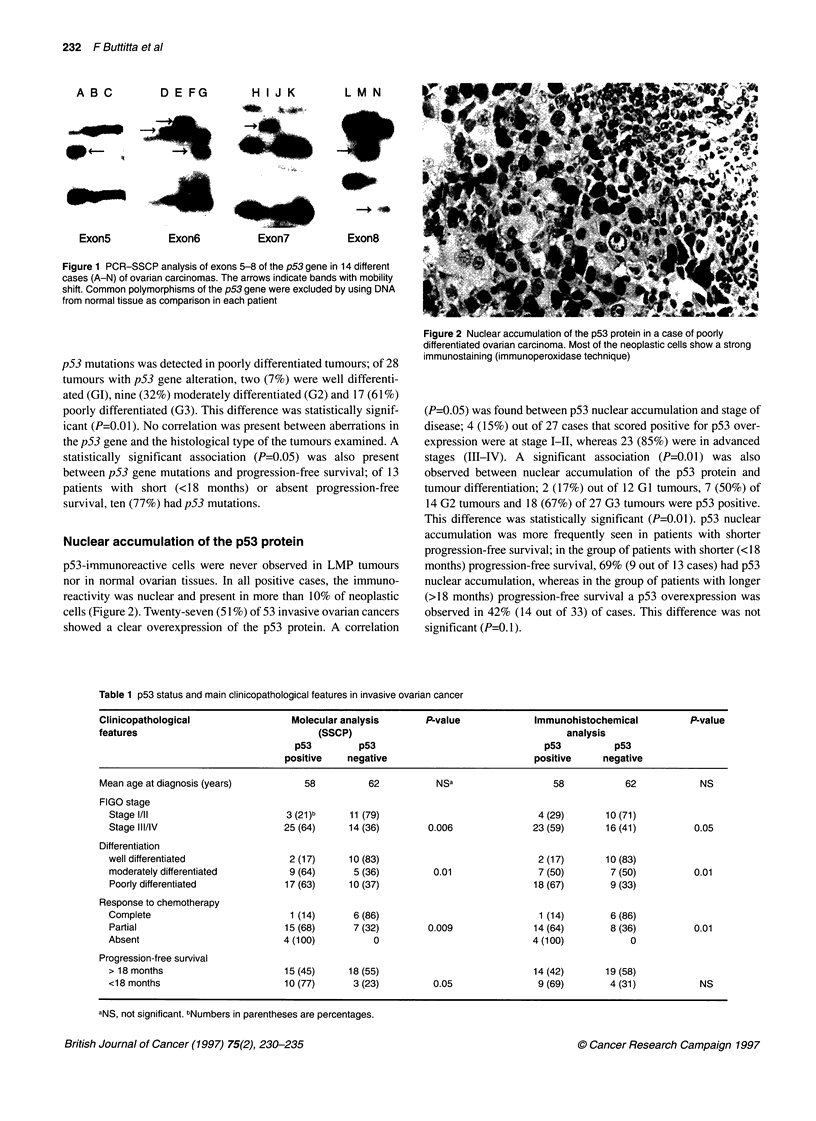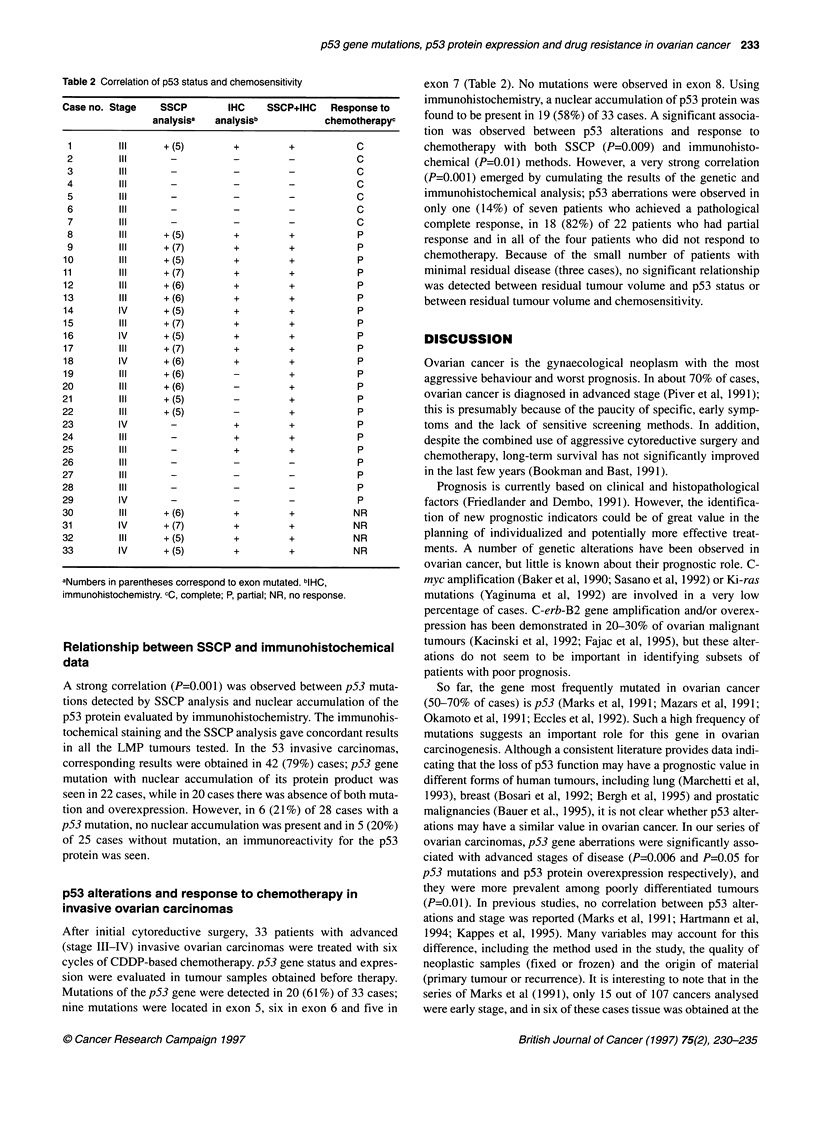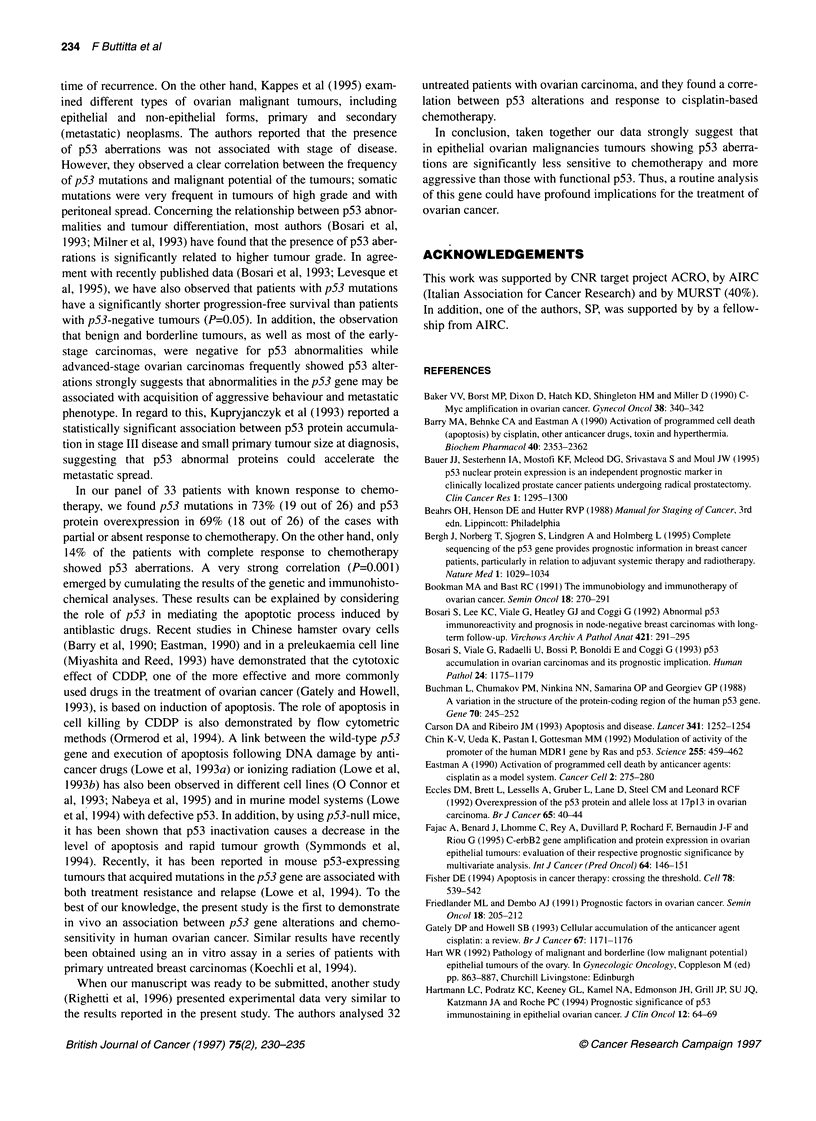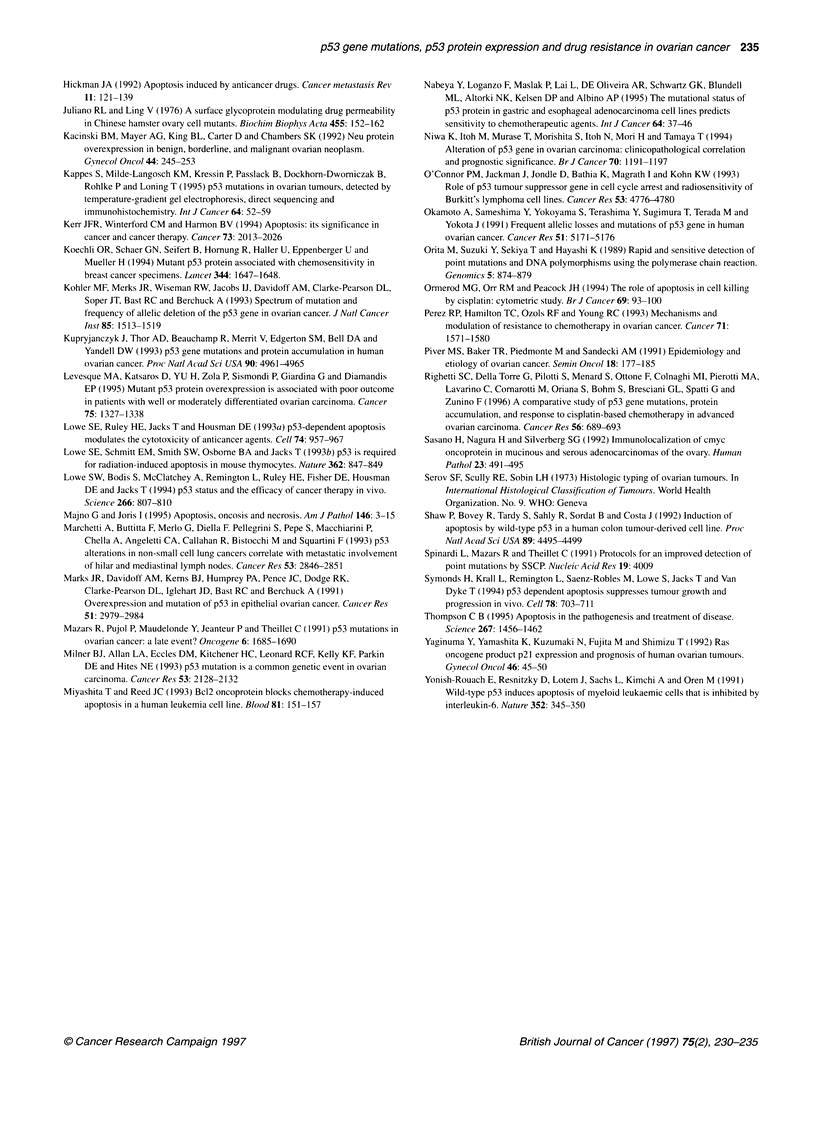# p53 alterations are predictive of chemoresistance and aggressiveness in ovarian carcinomas: a molecular and immunohistochemical study.

**DOI:** 10.1038/bjc.1997.38

**Published:** 1997

**Authors:** F. Buttitta, A. Marchetti, A. Gadducci, S. Pellegrini, M. Morganti, V. Carnicelli, S. Cosio, O. Gagetti, A. R. Genazzani, G. Bevilacqua

**Affiliations:** Institute of Pathology, University of Pisa, Italy.

## Abstract

**Images:**


British Journal of Cancer (1997) 75(2), 230–235
© 1997 Cancer Research Campaign

### p53 alterations are predictive of chemoresistance and aggressiveness in ovarian carcinomas: a molecular and immunohistochemical study

**F Buttitta[1], A Marchetti[1], A Gadducci[2], S Pellegrini[1], M Morganti[1], V Carnicelli[1], S Cosio[2], O Gagetti[2], AR Genazzani[2] and G Bevilacqua[1]**

[1]Institute of Pathology and [2]Department of Obstetrics and Gynaecology, University of Pisa, via Roma 57, 56100 Pisa, Italy

**Summary** Chemotherapeutic management of ovarian cancers is a difficult task as these neoplasms show significant differences in chemosensitivity, even if they share identical clinicopathological features. The present study was undertaken to investigate the prognostic and predictive role of p53 alterations in ovarian cancer. To this end, using different technical approaches, i.e. genetic and immunohistochemical analyses, we analysed a series of 68 ovarian neoplasms including 15 low malignant potential (LMP) tumours and 53 invasive carcinomas. We never observed p53 abnormalities in LMP tumours. p53 alterations were present only in invasive ovarian carcinomas, and they were detected much more frequently in tumours characterized by high histological grade ($P$=0.01) and advanced-stage disease ($P$=0.006 and $P$=0.05 for gene mutations and protein expression respectively). For 33 patients with invasive ovarian cancer, information was available concerning response to cisplatin-based chemotherapy. A strong correlation ($P$=0.001) has emerged between p53 alterations and response to chemotherapy: only one (14%) of seven patients who had a pathological complete response to antiblastic drugs showed p53 aberrations, whereas 18 (82%) of 22 cases with partial response and all of the four non-responsive patients scored positive for p53 abnormalities. We also observed that patients with *p53* mutations had a significantly shorter progression-free survival than patients with *p53*-negative tumours ($P$=0.05). Taken together, our results strongly suggest that in epithelial ovarian malignancies tumours showing p53 aberrations are significantly less sensitive to chemotherapy and more aggressive than those with functional *p53*. Thus, a routine analysis of this gene could have profound implications for the treatment of ovarian cancer.

**Keywords:** p53 mutations; p53 expression; polymerase chain reaction; single-strand conformation polymorphism; immunohistochemistry; ovarian carcinoma; *cis*-diamminedichloroplatinum II (cisplatin); chemoresistance

In most malignancies, drug resistance represents a major impediment to the control of neoplastic growth. Ovarian cancer presents an example of difficult chemotherapeutic management. Despite the improvements in therapeutic response, obtained mainly with *cis*-diamminedichloroplatinum II (CDDP)-based chemotherapy, approximately 50% of patients in advanced stage of disease are intrinsically resistant to chemotherapy and nearly 50% of originally responsive patients develop chemoresistance during the course of their treatment (Perez et al, 1993). In addition, ovarian cancers may show significant differences in chemosensitivity, even if they share identical clinicopathological features.

An increasing body of evidence suggests that most anti-cancer agents commonly used in the treatment of several malignancies may induce tumour regression by apoptosis (Hickman, 1992; Carson and Ribeiro, 1993; Fisher, 1994; Thompson, 1995), a peculiar form of cell death with biochemical and morphological features distinct from those of necrosis (Majno and Joris, 1995).

The apoptotic process is modulated by some proto-oncogenes and tumour-suppressor genes (Kerr et al, 1994; Thompson, 1995). Among the latter, the *p53* gene seems to have a crucial role in the execution of some forms of apoptosis (Yonish-Rouach et al, 1991; Shaw et al, 1992; Kerr et al, 1994). It has been shown in vitro that loss of *p53* function, through mutations that interfere with apoptosis, facilitates the development of neoplastic clones (Symonds et al, 1994) resistant to different antiblastic drugs (Lowe et al, 1993*a*) including CDDP (Nabeya et al, 1995). In addition, *p53* mutations could exert a specific activation of the MDR1 (multiple drug resistance) gene promoter (Chin et al, 1992), which may result in a reduced intracellular concentration of various chemotherapeutic agents (Juliano and Ling, 1976).

p53 alterations are common genetic events in ovarian carcinomas (Kohler et al, 1993; Kupryjanczyk et al, 1993; Milner et al, 1993), but at the moment it is not clear whether they have a prognostic value (Bosari et al, 1993; Niwa et al, 1994; Kappes et al, 1995) and their role in chemoresistance has not been established.

The present study was undertaken to investigate the prognostic and predictive role of p53 alterations in ovarian cancer. To this end, we evaluated the status of the *p53* gene by genetic analysis and the expression of the p53 protein by immunohistochemistry in a series of 68 ovarian carcinomas. The results were compared with well-known clinicopathological parameters of prognosis and with response to antiblastic agents in a subset of 33 patients treated with CDDP-based chemotherapy.

*Received 26 March 1996*
*Revised 9 July 1996*
*Accepted 23 July 1996*

*Correspondence to:* F Buttitta

### MATERIALS AND METHODS

### Patients and tissue samples

Sixty-eight patients with ovarian neoplasm, including 15 with low malignant potential (LMP) tumours (Hart, 1992) and 53 with invasive ovarian carcinoma, were analysed in this study.

Tumour specimens were consecutively obtained at initial surgical resection and immediately frozen at –70°C. To overcome the limitations due to the heterogeneity of ovarian cancer, adjacent sections of tumour were obtained in each case for DNA extraction and for immunoperoxidase staining. In each case, segments of normal fallopian tube or peripheral blood were collected as controls.

The carcinomas were histologically typed and graded according to the World Health Organization (Serov et al, 1973). Only epithelial ovarian malignancies were included in this study; among the invasive tumours there were 31 serous, ten endometrioid, three mucinous, three clear cell and six undifferentiated carcinomas. All LMP tumours were classified as serous. With respect to grade, there were 12 well-differentiated (G1), 14 moderately (G2) and 27 poorly differentiated tumours (G3).

Tumour stage was determined according to the criteria of the International Federation of Gynaecology and Obstetrics (FIGO) (Beahrs et al, 1988). For statistical analysis, patients with stage I and stage II disease were pooled into an early stage subgroup, whereas patients with stage III and IV disease were pooled into an advanced stage subgroup; 14 of the patients under study had stage I–II disease and 39 patients had stage III–IV disease.

The age range of these patients was 41–81 years, with a median of 59 years.

Forty-six patients with follow-up data ranging from 8 to 92 months' duration were analysed for progression-free length of survival. The evaluation of the clinical course of disease was based on clinical examination, chest radiography, abdominal–pelvic ultrasound and computerized tomography (CT) scan. After initial surgery, 33 patients with stage III–IV invasive ovarian cancer received six cycles of CDDP-based chemotherapy. The disease progressed or remained stable in four patients. The other 29 patients who achieved a clinical complete or partial response to chemotherapy underwent a second-look laparotomy. A pathological complete response to therapy was defined as the disappearance of all tumour deposits with negative peritoneal washing and negative multiple random biopsies.

### Detection of p53 alterations by SSCP

Polymerase chain reaction–single-strand conformation polymorphism (PCR–SSCP) analysis (Orita et al, 1989) was performed to detect mutations in the exon 5–8 of the *p53* gene. Samples were subjected to amplification at each exon, including the exon–intron boundary. The technique was optimized as previously described (Spinardi et al, 1991). Routinely, 100 ng of genomic DNA was used in a 10-μl PCR reaction containing 10 mM Tris-HCl (pH8.3), 1.5 mM magnesium chloride, 50 mM potassium chloride, 0.01% (w/v) gelatine, 1.25 mM each of four dNTPs (Boehringer Mannheim Biochemica), 1 mM of each primer, 0.5 μl of [α-$^{32}$P]dCTP (3000 Ci mmol$^{-1}$, Amersham, Arlington, IL, USA) and 0.25 units of *Taq* DNA polymerase (Perkin-Elmer Cetus, Norwalk, CT, USA). Four couples of specific intronic primers, whose sequences were deduced from Buchman et al (1988), were used:

Exon 5:
Sense: 5′-TGACTTTCAACTCTGTCTCCT
Anti: 5′-TCAGTGAGGAATGAGAGGCC

Exon 6:
Sense: 5′-CTGGAGAGACGACAGGGCTG
Anti: 5′-CCAFAFACCCCAGTTGCAAAC

Exon 7:
Sense: 5′-CTCGCGCACTGGCCTCATCTT
Anti: 5′-TCAGCGGCAAGCAGAGGCTG

Exon 8:
Sense: 5′-GGACAGGTAGGACCTGATTTCCTTAC
Anti: 5′-TGCACCCTTGGTCTCCTCCAC

Thirty amplification cycles were performed under the following conditions: initial denaturation, 6 min at 94°C; amplification, 1 min at 94°C, 1 min at 58°C, 2 min at 72°C for 30 cycles; elongation, 5 min at 72°C.

PCR products were electrophoretically separated by 6% non-denaturing polyacrylamide gel at 10 W for 12 h at 20°C. Genomic DNAs from tumour samples and corresponding normal ovarian tissues were examined by separate PCR–SSCP analysis to exclude the possibility of any polymorphism within the coding region. Upon complete migration, the gels were dried and subjected to autoradiography against a Kodak XAR-5 film at –80°C with an intensifying screen. PCR was performed at least twice for each sample, and only the reproducible cases were taken.

### Immunohistochemical analysis

Immunostaining of the p53 protein was performed by the avidin–biotin peroxidase complex method. Briefly, 4–5 μm frozen sections were cut, mounted on polylysine-coated slides, air dried and fixed in cold (4°C) acetone for 10 min before being subsequently incubated with: (1) monoclonal antibody 1801 (Ab-2; Oncogene Science, Vector, Burlingame, CA, USA) (diluted 1:100, overnight at 4°C) which recognizes a denaturation-resistant epitope from amino acid 32 to 79 of the p53 protein; (2) a biotinylated horse anti-mouse IgG antibody (diluted 1:500, 30 min at room temperature); (3) avidin–peroxidase complexes (1 h). Finally, the slides were developed with 0.5% diaminobenzidine in 0.05 M Tris buffer pH 7.4 containing 0.5% hydrogen peroxide, rinsed in tap water, counterstained with 5% haematoxylin, dehydrated, cleared in xylene and mounted in permanent coverslipping medium. Positive controls were two known positive cases of human colon cancer. Negative controls were obtained by replacement of primary antiserum with Tris buffer.

### Statistical analysis

Contingency tables were used to examine the relationship between aberrations of the *p53* gene and each of the following clinicopathological data: stage of disease, residual disease, grade of tumour differentiation, response to chemotherapy and progression-free overall survival. Statistical association was determined by $\chi^2$ analysis. A *P*-value of less than 0.05 was considered to have statistical significance.

### RESULTS

### *p53* gene mutations

In all the LMP tumours tested, no *p53* gene mutations were found. Electrophoretic mobility shifts, indicative of mutation of *p53*, were detected in 28 (53%) of 53 invasive carcinomas; 11 mutations were located in exon 5, six in exon 6, eight in exon 7 and three in exon 8 (Figure 1). A strong correlation (*P*=0.006) was observed between *p53* gene mutations and FIGO stage; *p53* mutations were found in 3 (21%) of 14 tumours at stage I–II and in 25 (64%) of 39 cases at stage III–IV (Table 1). A higher frequency of

© Cancer Research Campaign 1997

**Figure 1** PCR–SSCP analysis of exons 5–8 of the *p53* gene in 14 different cases (A–N) of ovarian carcinomas. The arrows indicate bands with mobility shift. Common polymorphisms of the *p53* gene were excluded by using DNA from normal tissue as comparison in each patient

**Figure 2** Nuclear accumulation of the p53 protein in a case of poorly differentiated ovarian carcinoma. Most of the neoplastic cells show a strong immunostaining (immunoperoxidase technique)

*p53* mutations was detected in poorly differentiated tumours; of 28 tumours with *p53* gene alteration, two (7%) were well differentiated (GI), nine (32%) moderately differentiated (G2) and 17 (61%) poorly differentiated (G3). This difference was statistically significant ($P$=0.01). No correlation was present between aberrations in the *p53* gene and the histological type of the tumours examined. A statistically significant association ($P$=0.05) was also present between *p53* gene mutations and progression-free survival; of 13 patients with short (<18 months) or absent progression-free survival, ten (77%) had *p53* mutations.

### Nuclear accumulation of the p53 protein p53-immunoreactive cells were never observed in LMP tumours nor in normal ovarian tissues. In all positive cases, the immunoreactivity was nuclear and present in more than 10% of neoplastic cells (Figure 2). Twenty-seven (51%) of 53 invasive ovarian cancers showed a clear overexpression of the p53 protein. A correlation ($P$=0.05) was found between p53 nuclear accumulation and stage of disease; 4 (15%) out of 27 cases that scored positive for p53 overexpression were at stage I–II, whereas 23 (85%) were in advanced stages (III–IV). A significant association ($P$=0.01) was also observed between nuclear accumulation of the p53 protein and tumour differentiation; 2 (17%) out of 12 G1 tumours, 7 (50%) of 14 G2 tumours and 18 (67%) of 27 G3 tumours were p53 positive. This difference was statistically significant ($P$=0.01). p53 nuclear accumulation was more frequently seen in patients with shorter progression-free survival; in the group of patients with shorter (<18 months) progression-free survival, 69% (9 out of 13 cases) had p53 nuclear accumulation, whereas in the group of patients with longer (>18 months) progression-free survival a p53 overexpression was observed in 42% (14 out of 33) of cases. This difference was not significant ($P$=0.1).

**Table 1** p53 status and main clinicopathological features in invasive ovarian cancer

| Clinicopathological features | Molecular analysis (SSCP) | | *P*-value | Immunohistochemical analysis | | *P*-value |
|---|---|---|---|---|---|---|
| | p53 positive | p53 negative | | p53 positive | p53 negative | |
| Mean age at diagnosis (years) | 58 | 62 | NS[a] | 58 | 62 | NS |
| FIGO stage | | | | | | |
| Stage I/II | 3 (21)[b] | 11 (79) | | 4 (29) | 10 (71) | |
| Stage III/IV | 25 (64) | 14 (36) | 0.006 | 23 (59) | 16 (41) | 0.05 |
| Differentiation | | | | | | |
| well differentiated | 2 (17) | 10 (83) | | 2 (17) | 10 (83) | |
| moderately differentiated | 9 (64) | 5 (36) | 0.01 | 7 (50) | 7 (50) | 0.01 |
| Poorly differentiated | 17 (63) | 10 (37) | | 18 (67) | 9 (33) | |
| Response to chemotherapy | | | | | | |
| Complete | 1 (14) | 6 (86) | | 1 (14) | 6 (86) | |
| Partial | 15 (68) | 7 (32) | 0.009 | 14 (64) | 8 (36) | 0.01 |
| Absent | 4 (100) | 0 | | 4 (100) | 0 | |
| Progression-free survival | | | | | | |
| > 18 months | 15 (45) | 18 (55) | | 14 (42) | 19 (58) | |
| <18 months | 10 (77) | 3 (23) | 0.05 | 9 (69) | 4 (31) | NS |

[a]NS, not significant. [b]Numbers in parentheses are percentages.

 © Cancer Research Campaign 1997

**Table 2** Correlation of p53 status and chemosensitivity

| Case no. | Stage | SSCP analysis[a] | IHC analysis[b] | SSCP+IHC | Response to chemotherapy[c] |
|---|---|---|---|---|---|
| 1 | III | + (5) | + | + | C |
| 2 | III | – | – | – | C |
| 3 | III | – | – | – | C |
| 4 | III | – | – | – | C |
| 5 | III | – | – | – | C |
| 6 | III | – | – | – | C |
| 7 | III | – | – | – | C |
| 8 | III | + (5) | + | + | P |
| 9 | III | + (7) | + | + | P |
| 10 | III | + (5) | + | + | P |
| 11 | III | + (7) | + | + | P |
| 12 | III | + (6) | + | + | P |
| 13 | III | + (6) | + | + | P |
| 14 | IV | + (5) | + | + | P |
| 15 | III | + (7) | + | + | P |
| 16 | IV | + (5) | + | + | P |
| 17 | III | + (7) | + | + | P |
| 18 | IV | + (6) | + | + | P |
| 19 | III | + (6) | – | + | P |
| 20 | III | + (6) | – | + | P |
| 21 | III | + (5) | – | + | P |
| 22 | III | + (5) | – | + | P |
| 23 | IV | – | + | + | P |
| 24 | III | – | + | + | P |
| 25 | III | – | + | + | P |
| 26 | III | – | – | – | P |
| 27 | III | – | – | – | P |
| 28 | III | – | – | – | P |
| 29 | IV | – | – | – | P |
| 30 | III | + (6) | + | + | NR |
| 31 | IV | + (7) | + | + | NR |
| 32 | III | + (5) | + | + | NR |
| 33 | IV | + (5) | + | + | NR |

[a]Numbers in parentheses correspond to exon mutated. [b]IHC, immunohistochemistry. [c]C, complete; P, partial; NR, no response.

### Relationship between SSCP and immunohistochemical data

A strong correlation ($P$=0.001) was observed between *p53* mutations detected by SSCP analysis and nuclear accumulation of the p53 protein evaluated by immunohistochemistry. The immunohistochemical staining and the SSCP analysis gave concordant results in all the LMP tumours tested. In the 53 invasive carcinomas, corresponding results were obtained in 42 (79%) cases; *p53* gene mutation with nuclear accumulation of its protein product was seen in 22 cases, while in 20 cases there was absence of both mutation and overexpression. However, in 6 (21%) of 28 cases with a *p53* mutation, no nuclear accumulation was present and in 5 (20%) of 25 cases without mutation, an immunoreactivity for the p53 protein was seen.

### p53 alterations and response to chemotherapy in invasive ovarian carcinomas

After initial cytoreductive surgery, 33 patients with advanced (stage III–IV) invasive ovarian carcinomas were treated with six cycles of CDDP-based chemotherapy. *p53* gene status and expression were evaluated in tumour samples obtained before therapy. Mutations of the *p53* gene were detected in 20 (61%) of 33 cases; nine mutations were located in exon 5, six in exon 6 and five in exon 7 (Table 2). No mutations were observed in exon 8. Using immunohistochemistry, a nuclear accumulation of p53 protein was found to be present in 19 (58%) of 33 cases. A significant association was observed between p53 alterations and response to chemotherapy with both SSCP ($P$=0.009) and immunohistochemical ($P$=0.01) methods. However, a very strong correlation ($P$=0.001) emerged by cumulating the results of the genetic and immunohistochemical analysis; p53 aberrations were observed in only one (14%) of seven patients who achieved a pathological complete response, in 18 (82%) of 22 patients who had partial response and in all of the four patients who did not respond to chemotherapy. Because of the small number of patients with minimal residual disease (three cases), no significant relationship was detected between residual tumour volume and p53 status or between residual tumour volume and chemosensitivity.

### DISCUSSION

Ovarian cancer is the gynaecological neoplasm with the most aggressive behaviour and worst prognosis. In about 70% of cases, ovarian cancer is diagnosed in advanced stage (Piver et al, 1991); this is presumably because of the paucity of specific, early symptoms and the lack of sensitive screening methods. In addition, despite the combined use of aggressive cytoreductive surgery and chemotherapy, long-term survival has not significantly improved in the last few years (Bookman and Bast, 1991).

Prognosis is currently based on clinical and histopathological factors (Friedlander and Dembo, 1991). However, the identification of new prognostic indicators could be of great value in the planning of individualized and potentially more effective treatments. A number of genetic alterations have been observed in ovarian cancer, but little is known about their prognostic role. C-*myc* amplification (Baker et al, 1990; Sasano et al, 1992) or Ki-*ras* mutations (Yaginuma et al, 1992) are involved in a very low percentage of cases. C-*erb*-B2 gene amplification and/or overexpression has been demonstrated in 20–30% of ovarian malignant tumours (Kacinski et al, 1992; Fajac et al, 1995), but these alterations do not seem to be important in identifying subsets of patients with poor prognosis.

So far, the gene most frequently mutated in ovarian cancer (50–70% of cases) is *p53* (Marks et al, 1991; Mazars et al, 1991; Okamoto et al, 1991; Eccles et al, 1992). Such a high frequency of mutations suggests an important role for this gene in ovarian carcinogenesis. Although a consistent literature provides data indicating that the loss of p53 function may have a prognostic value in different forms of human tumours, including lung (Marchetti et al, 1993), breast (Bosari et al, 1992; Bergh et al, 1995) and prostatic malignancies (Bauer et al., 1995), it is not clear whether p53 alterations may have a similar value in ovarian cancer. In our series of ovarian carcinomas, *p53* gene aberrations were significantly associated with advanced stages of disease ($P$=0.006 and $P$=0.05 for *p53* mutations and p53 protein overexpression respectively), and they were more prevalent among poorly differentiated tumours ($P$=0.01). In previous studies, no correlation between p53 alterations and stage was reported (Marks et al, 1991; Hartmann et al, 1994; Kappes et al, 1995). Many variables may account for this difference, including the method used in the study, the quality of neoplastic samples (fixed or frozen) and the origin of material (primary tumour or recurrence). It is interesting to note that in the series of Marks et al (1991), only 15 out of 107 cancers analysed were early stage, and in six of these cases tissue was obtained at the

© Cancer Research Campaign 1997

time of recurrence. On the other hand, Kappes et al (1995) examined different types of ovarian malignant tumours, including epithelial and non-epithelial forms, primary and secondary (metastatic) neoplasms. The authors reported that the presence of p53 aberrations was not associated with stage of disease. However, they observed a clear correlation between the frequency of *p53* mutations and malignant potential of the tumours; somatic mutations were very frequent in tumours of high grade and with peritoneal spread. Concerning the relationship between p53 abnormalities and tumour differentiation, most authors (Bosari et al, 1993; Milner et al, 1993) have found that the presence of p53 aberrations is significantly related to higher tumour grade. In agreement with recently published data (Bosari et al, 1993; Levesque et al, 1995), we have also observed that patients with *p53* mutations have a significantly shorter progression-free survival than patients with *p53*-negative tumours ($P$=0.05). In addition, the observation that benign and borderline tumours, as well as most of the early-stage carcinomas, were negative for p53 abnormalities while advanced-stage ovarian carcinomas frequently showed p53 alterations strongly suggests that abnormalities in the *p53* gene may be associated with acquisition of aggressive behaviour and metastatic phenotype. In regard to this, Kupryjanczyk et al (1993) reported a statistically significant association between p53 protein accumulation in stage III disease and small primary tumour size at diagnosis, suggesting that p53 abnormal proteins could accelerate the metastatic spread.

In our panel of 33 patients with known response to chemotherapy, we found *p53* mutations in 73% (19 out of 26) and p53 protein overexpression in 69% (18 out of 26) of the cases with partial or absent response to chemotherapy. On the other hand, only 14% of the patients with complete response to chemotherapy showed p53 aberrations. A very strong correlation ($P$=0.001) emerged by cumulating the results of the genetic and immunohistochemical analyses. These results can be explained by considering the role of *p53* in mediating the apoptotic process induced by antiblastic drugs. Recent studies in Chinese hamster ovary cells (Barry et al, 1990; Eastman, 1990) and in a preleukaemia cell line (Miyashita and Reed, 1993) have demonstrated that the cytotoxic effect of CDDP, one of the more effective and more commonly used drugs in the treatment of ovarian cancer (Gately and Howell, 1993), is based on induction of apoptosis. The role of apoptosis in cell killing by CDDP is also demonstrated by flow cytometric methods (Ormerod et al, 1994). A link between the wild-type *p53* gene and execution of apoptosis following DNA damage by anticancer drugs (Lowe et al, 1993*a*) or ionizing radiation (Lowe et al, 1993*b*) has also been observed in different cell lines (O Connor et al, 1993; Nabeya et al, 1995) and in murine model systems (Lowe et al, 1994) with defective p53. In addition, by using *p53*-null mice, it has been shown that p53 inactivation causes a decrease in the level of apoptosis and rapid tumour growth (Symmonds et al, 1994). Recently, it has been reported in mouse p53-expressing tumours that acquired mutations in the *p53* gene are associated with both treatment resistance and relapse (Lowe et al, 1994). To the best of our knowledge, the present study is the first to demonstrate in vivo an association between *p53* gene alterations and chemosensitivity in human ovarian cancer. Similar results have recently been obtained using an in vitro assay in a series of patients with primary untreated breast carcinomas (Koechli et al, 1994).

When our manuscript was ready to be submitted, another study (Righetti et al, 1996) presented experimental data very similar to the results reported in the present study. The authors analysed 32 untreated patients with ovarian carcinoma, and they found a correlation between p53 alterations and response to cisplatin-based chemotherapy.

In conclusion, taken together our data strongly suggest that in epithelial ovarian malignancies tumours showing p53 aberrations are significantly less sensitive to chemotherapy and more aggressive than those with functional p53. Thus, a routine analysis of this gene could have profound implications for the treatment of ovarian cancer.

### ACKNOWLEDGEMENTS

This work was supported by CNR target project ACRO, by AIRC (Italian Association for Cancer Research) and by MURST (40%). In addition, one of the authors, SP, was supported by by a fellowship from AIRC.

### REFERENCES

Baker VV, Borst MP, Dixon D, Hatch KD, Shingleton HM and Miller D (1990) C-Myc amplification in ovarian cancer. *Gynecol Oncol* **38**: 340–342

Barry MA, Behnke CA and Eastman A (1990) Activation of programmed cell death (apoptosis) by cisplatin, other anticancer drugs, toxin and hyperthermia. *Biochem Pharmacol* **40**: 2353–2362

Bauer JJ, Sesterhenn IA, Mostofi KF, Mcleod DG, Srivastava S and Moul JW (1995) p53 nuclear protein expression is an independent prognostic marker in clinically localized prostate cancer patients undergoing radical prostatectomy. *Clin Cancer Res* **1**: 1295–1300

Beahrs OH, Henson DE and Hutter RVP (1988) *Manual for Staging of Cancer*, 3rd edn. Lippincott: Philadelphia

Bergh J, Norberg T, Sjogren S, Lindgren A and Holmberg L (1995) Complete sequencing of the p53 gene provides prognostic information in breast cancer patients, particularly in relation to adjuvant systemic therapy and radiotherapy. *Nature Med* **1**: 1029–1034

Bookman MA and Bast RC (1991) The immunobiology and immunotherapy of ovarian cancer. *Semin Oncol* **18**: 270–291

Bosari S, Lee KC, Viale G, Heatley GJ and Coggi G (1992) Abnormal p53 immunoreactivity and prognosis in node-negative breast carcinomas with long-term follow-up. *Virchows Archiv A Pathol Anat* **421**: 291–295

Bosari S, Viale G, Radaelli U, Bossi P, Bonoldi E and Coggi G (1993) p53 accumulation in ovarian carcinomas and its prognostic implication. *Human Pathol* **24**: 1175–1179

Buchman L, Chumakov PM, Ninkina NN, Samarina OP and Georgiev GP (1988) A variation in the structure of the protein-coding region of the human p53 gene. *Gene* **70**: 245–252

Carson DA and Ribeiro JM (1993) Apoptosis and disease. *Lancet* **341**: 1252–1254

Chin K-V, Ueda K, Pastan I, Gottesman MM (1992) Modulation of activity of the promoter of the human MDR1 gene by Ras and p53. *Science* **255**: 459–462

Eastman A (1990) Activation of programmed cell death by anticancer agents: cisplatin as a model system. *Cancer Cell* **2**: 275–280

Eccles DM, Brett L, Lessells A, Gruber L, Lane D, Steel CM and Leonard RCF (1992) Overexpression of the p53 protein and allele loss at 17p13 in ovarian carcinoma. *Br J Cancer* **65**: 40–44

Fajac A, Benard J, Lhomme C, Rey A, Duvillard P, Rochard F, Bernaudin J-F and Riou G (1995) C-erbB2 gene amplification and protein expression in ovarian epithelial tumours: evaluation of their respective prognostic significance by multivariate analysis. *Int J Cancer (Pred Oncol)* **64**: 146–151

Fisher DE (1994) Apoptosis in cancer therapy: crossing the threshold. *Cell* **78**: 539–542

Friedlander ML and Dembo AJ (1991) Prognostic factors in ovarian cancer. *Semin Oncol* **18**: 205–212

Gately DP and Howell SB (1993) Cellular accumulation of the anticancer agent cisplatin: a review. *Br J Cancer* **67**: 1171–1176

Hart WR (1992) Pathology of malignant and borderline (low malignant potential) epithelial tumours of the ovary. In *Gynecologic Oncology*, Coppleson M (ed) pp. 863–887, Churchill Livingstone: Edinburgh

Hartmann LC, Podratz KC, Keeney GL, Kamel NA, Edmonson JH, Grill JP, SU JQ, Katzmann JA and Roche PC (1994) Prognostic significance of p53 immunostaining in epithelial ovarian cancer. *J Clin Oncol* **12**: 64–69

© Cancer Research Campaign 1997

Hickman JA (1992) Apoptosis induced by anticancer drugs. *Cancer metastasis Rev* **11**: 121–139

Juliano RL and Ling V (1976) A surface glycoprotein modulating drug permeability in Chinese hamster ovary cell mutants. *Biochim Biophys Acta* **455**: 152–162

Kacinski BM, Mayer AG, King BL, Carter D and Chambers SK (1992) Neu protein overexpression in benign, borderline, and malignant ovarian neoplasm. *Gynecol Oncol* **44**: 245–253

Kappes S, Milde-Langosch KM, Kressin P, Passlack B, Dockhorn-Dworniczak B, Rohlke P and Loning T (1995) p53 mutations in ovarian tumours, detected by temperature-gradient gel electrophoresis, direct sequencing and immunohistochemistry. *Int J Cancer* **64**: 52–59

Kerr JFR, Winterford CM and Harmon BV (1994) Apoptosis: its significance in cancer and cancer therapy. *Cancer* **73**: 2013–2026

Koechli OR, Schaer GN, Seifert B, Hornung R, Haller U, Eppenberger U and Mueller H (1994) Mutant p53 protein associated with chemosensitivity in breast cancer specimens. *Lancet* **344**: 1647–1648.

Kohler MF, Merks JR, Wiseman RW, Jacobs IJ, Davidoff AM, Clarke-Pearson DL, Soper JT, Bast RC and Berchuck A (1993) Spectrum of mutation and frequency of allelic deletion of the p53 gene in ovarian cancer. *J Natl Cancer Inst* **85**: 1513–1519

Kupryjanczyk J, Thor AD, Beauchamp R, Merrit V, Edgerton SM, Bell DA and Yandell DW (1993) p53 gene mutations and protein accumulation in human ovarian cancer. *Proc Natl Acad Sci USA* **90**: 4961–4965

Levesque MA, Katsaros D, YU H, Zola P, Sismondi P, Giardina G and Diamandis EP (1995) Mutant p53 protein overexpression is associated with poor outcome in patients with well or moderately differentiated ovarian carcinoma. *Cancer* **75**: 1327–1338

Lowe SE, Ruley HE, Jacks T and Housman DE (1993*a*) p53-dependent apoptosis modulates the cytotoxicity of anticancer agents. *Cell* **74**: 957–967

Lowe SE, Schmitt EM, Smith SW, Osborne BA and Jacks T (1993*b*) p53 is required for radiation-induced apoptosis in mouse thymocytes. *Nature* **362**: 847–849

Lowe SW, Bodis S, McClatchey A, Remington L, Ruley HE, Fisher DE, Housman DE and Jacks T (1994) p53 status and the efficacy of cancer therapy in vivo. *Science* **266**: 807–810

Majno G and Joris I (1995) Apoptosis, oncosis and necrosis. *Am J Pathol* **146**: 3–15

Marchetti A, Buttitta F, Merlo G, Diella F. Pellegrini S, Pepe S, Macchiarini P, Chella A, Angeletti CA, Callahan R, Bistocchi M and Squartini F (1993) p53 alterations in non-small cell lung cancers correlate with metastatic involvement of hilar and mediastinal lymph nodes. *Cancer Res* **53**: 2846–2851

Marks JR, Davidoff AM, Kerns BJ, Humprey PA, Pence JC, Dodge RK, Clarke-Pearson DL, Iglehart JD, Bast RC and Berchuck A (1991) Overexpression and mutation of p53 in epithelial ovarian cancer. *Cancer Res* **51**: 2979–2984

Mazars R, Pujol P, Maudelonde Y, Jeanteur P and Theillet C (1991) p53 mutations in ovarian cancer: a late event? *Oncogene* **6**: 1685–1690

Milner BJ, Allan LA, Eccles DM, Kitchener HC, Leonard RCF, Kelly KF, Parkin DE and Hites NE (1993) p53 mutation is a common genetic event in ovarian carcinoma. *Cancer Res* **53**: 2128–2132

Miyashita T and Reed JC (1993) Bcl2 oncoprotein blocks chemotherapy-induced apoptosis in a human leukemia cell line. *Blood* **81**: 151–157

Nabeya Y, Loganzo F, Maslak P, Lai L, DE Oliveira AR, Schwartz GK, Blundell ML, Altorki NK, Kelsen DP and Albino AP (1995) The mutational status of p53 protein in gastric and esophageal adenocarcinoma cell lines predicts sensitivity to chemotherapeutic agents. *Int J Cancer* **64**: 37–46

Niwa K, Itoh M, Murase T, Morishita S, Itoh N, Mori H and Tamaya T (1994) Alteration of p53 gene in ovarian carcinoma: clinicopathological correlation and prognostic significance. *Br J Cancer* **70**: 1191–1197

O'Connor PM, Jackman J, Jondle D, Bathia K, Magrath I and Kohn KW (1993) Role of p53 tumour suppressor gene in cell cycle arrest and radiosensitivity of Burkitt's lymphoma cell lines. *Cancer Res* **53**: 4776–4780

Okamoto A, Sameshima Y, Yokoyama S, Terashima Y, Sugimura T, Terada M and Yokota J (1991) Frequent allelic losses and mutations of p53 gene in human ovarian cancer. *Cancer Res* **51**: 5171–5176

Orita M, Suzuki Y, Sekiya T and Hayashi K (1989) Rapid and sensitive detection of point mutations and DNA polymorphisms using the polymerase chain reaction. *Genomics* **5**: 874–879

Ormerod MG, Orr RM and Peacock JH (1994) The role of apoptosis in cell killing by cisplatin: cytometric study. *Br J Cancer* **69**: 93–100

Perez RP, Hamilton TC, Ozols RF and Young RC (1993) Mechanisms and modulation of resistance to chemotherapy in ovarian cancer. *Cancer* **71**: 1571–1580

Piver MS, Baker TR, Piedmonte M and Sandecki AM (1991) Epidemiology and etiology of ovarian cancer. *Semin Oncol* **18**: 177–185

Righetti SC, Della Torre G, Pilotti S, Menard S, Ottone F, Colnaghi MI, Pierotti MA, Lavarino C, Cornarotti M, Oriana S, Bohm S, Bresciani GL, Spatti G and Zunino F (1996) A comparative study of p53 gene mutations, protein accumulation, and response to cisplatin-based chemotherapy in advanced ovarian carcinoma. *Cancer Res* **56**: 689–693

Sasano H, Nagura H and Silverberg SG (1992) Immunolocalization of cmyc oncoprotein in mucinous and serous adenocarcinomas of the ovary. *Human Pathol* **23**: 491–495

Serov SF, Scully RE, Sobin LH (1973) Histologic typing of ovarian tumours. In *International Histological Classification of Tumours*. World Health Organization. No. 9. WHO: Geneva

Shaw P, Bovey R, Tardy S, Sahly R, Sordat B and Costa J (1992) Induction of apoptosis by wild-type p53 in a human colon tumour-derived cell line. *Proc Natl Acad Sci USA* **89**: 4495–4499

Spinardi L, Mazars R and Theillet C (1991) Protocols for an improved detection of point mutations by SSCP. *Nucleic Acid Res* **19**: 4009

Symonds H, Krall L, Remington L, Saenz-Robles M, Lowe S, Jacks T and Van Dyke T (1994) p53 dependent apoptosis suppresses tumour growth and progression in vivo. *Cell* **78**: 703–711

Thompson C B (1995) Apoptosis in the pathogenesis and treatment of disease. *Science* **267**: 1456–1462

Yaginuma Y, Yamashita K, Kuzumaki N, Fujita M and Shimizu T (1992) Ras oncogene product p21 expression and prognosis of human ovarian tumours. *Gynecol Oncol* **46**: 45–50

Yonish-Rouach E, Resnitzky D, Lotem J, Sachs L, Kimchi A and Oren M (1991) Wild-type p53 induces apoptosis of myeloid leukaemic cells that is inhibited by interleukin-6. *Nature* **352**: 345–350

© Cancer Research Campaign 1997